# Fine motor skills in South African children with symptoms of ADHD: influence of subtype, gender, age, and hand dominance

**DOI:** 10.1186/1744-9081-2-33

**Published:** 2006-10-09

**Authors:** Anneke Meyer, Terje Sagvolden

**Affiliations:** 1 School of Health Sciences, University of Limpopo, Private Bag X1106, 0727 Sovenga, South Africa; 2 Department of Physiology, Institute of Basic Medical Sciences, University of Oslo, NO-0317 Oslo, Norway; 3 Centre for Advanced Study at the Norwegian Academy for Science and Letters, NO-0271 Oslo, Norway

## Abstract

**Background:**

Motor problems, often characterised as clumsiness or poor motor coordination, have been associated with ADHD in addition to the main symptom groups of inattention, impulsiveness, and overactivity. The problems addressed in this study were: (1) Are motor problems associated with ADHD symptoms, also in African cultures? (2) Are there differences in motor skills among the subtypes with ADHD symptoms? (3) Are there gender differences? (4) Is there an effect of age? (5) Are there differences in performance between the dominant and non-dominant hand?

**Method:**

A total of 528 children (264 classified as having symptoms of ADHD and 264 matched comparisons) of both genders and from seven different South African ethnic groups participated in the study. They were assessed with three simple, easy to administer instruments which measure various functions of motor speed and eye-hand coordination: The Grooved Pegboard, the Maze Coordination Task, and the Finger Tapping Test. The results were analysed as a function of subtype, gender, age, and hand dominance.

**Results:**

The findings indicate that children with symptoms of ADHD performed significantly poorer on the Grooved Pegboard and Motor Coordination Task, but not on the Finger Tapping Test than their comparisons without ADHD symptoms. The impairment was most severe for the subtype with symptoms of ADHD-C (combined) and less severe for the subtypes with symptoms of ADHD-PI (predominantly inattentive) and ADHD-HI (predominantly hyperactive/impulsive). With few exceptions, both genders were equally affected while there were only slight differences in performance between the dominant and non-dominant hand. The deficiencies in motor control were mainly confined to the younger age group (6 – 9 yr).

**Conclusion:**

An association between the symptoms of ADHD and motor problems was demonstrated in terms of accuracy and speed in fairly complex tasks, but not in simple motor tests of speed. This deficiency is found mainly in the children with ADHD-C symptoms, but also to a lesser degree in the children with symptoms of ADHD-PI and ADHD-HI.

## Background

Attention-Deficit/Hyperactivity Disorder (ADHD) is a prevalent, heterogeneous, and debilitating neurodevelopmental disorder with an early onset. The disorder consists of a persistent pattern of inattentiveness, impulsiveness, and/or hyperactivity that is inconsistent with the child's developmental level [1]. ADHD is the most common child psychiatric disorder in Europe and the United States, affecting 3–10% of primary school children [2-4]. The disorder is generally more prevalent in males, but more severe in females [5]. Male-to-female ratios vary from 9:1 to 6:1 in clinic-referred samples, but is only approximately 3:1 in population-based ones [3]. ADHD is associated with proneness for repeated accidents, depressive and anxiety disorders, learning disabilities, and school failure [4,6-9]. The disorder is also associated with later increased incidence of substance abuse if not diagnosed and treated at an early age [10]. Thus, the economic, educational, social, and personal costs of ADHD are enormous.

Although referral practices and assessment procedures are neither well developed nor standardized in developing countries [11], ADHD is as prevalent and has the same sex ratios on the African continent as in Western countries [12-14] suggesting that ADHD is caused by the same fundamental neurobiological factors probably hypofunctioning dopamine systems influencing among other functions learning and behavioural planning [15,16].

DSM-IV [1] identifies three subtypes of the disorder, namely: ADHD predominantly inattentive type (ADHD-PI) if six (or more) symptoms of inattention (but fewer than six symptoms of hyperactivity-impulsiveness) have persisted for at least 6 months; ADHD predominantly Hyperactive-Impulsive Type (ADHD-HI), if six (or more) symptoms of hyperactivity-impulsiveness (but fewer than six symptoms of inattention) have persisted for at least 6 months; and ADHD combined type (ADHD-C), if at least six symptoms of inattention and at least six symptoms of hyperactivity-impulsiveness have persisted for at least 6 months.

Besides the symptoms of inattention, hyperactivity, and impulsiveness, the ADHD child's motor ability is frequently significantly lower than would be expected of his age and level of intellectual functioning [1]. The wide range of motor problems include delays in achieving motor milestones, problems with movement planning and execution (reaction time, movement time, accuracy, and variability) [17], and parameter setting (not to be able to adapt their task performance to environmental requirements) [18]. Children with ADHD who experience motor problems often display deficits in requiring complex co-ordinations of movement, such as handwriting [2,18]. These problems may interfere with the ADHD child's daily functioning and influence their academic performance [19].

Development Coordination Disorder (DCD) is a disorder with marked impairment in the development of motor coordination which cannot be attributed to a general medical condition or mental retardation [1]. Although DSM-IV [1] does not link DCD with ADHD, the disorders co-occur in ~50% of cases [18,20,21] through a shared, additive genetic component between most subtypes of ADHD and DCD [22]. The children diagnosed with DCD who have problems with fine motor skills, are usually strongly linked to the ADHD-PI subtype [23]. In the Scandinavian countries the term DAMP (deficits in attention, motor control, and perception) is sometimes used for children with ADHD+DCD [8,24,25]. DAMP has been defined as a neurodevelopmental dysfunction syndrome with a high degree of comorbidity. Motor clumsiness is however, also associated with a variety of developmental disorders: learning disability [26], reading disorder [27], oppositional defiance disorder (ODD) [28] autism [17] and Asperger's syndrome [29,30].

The aspects of motor performance to be assessed in the present study were manual dexterity, complex coordination, and motor planning (by the Grooved Pegboard and the Maze Coordination Task), and manual dexterity (by the Finger Tapping Task).

The problems addressed were: Are motor problems associated with ADHD, also in African cultures? Are there differences in motor skills among the ADHD subtypes? Are there gender differences? Is there an effect of age? Are there differences in performance between the dominant and non-dominant hand?

## Method

### Sample

Children in the Limpopo Province of South Africa, consisting of 7 ethnic groups (Northern Sotho, Venda, Tsonga, Tswana, North Ndebele, Bolobedu, and Afrikaans) were recruited from a school-based population. The 528 children (378 boys and 150 girls) were recruited following screening for ADHD of the general population of primary school children representative of all socio-economic levels. Written permission was obtained from the Department of Education, Limpopo Province, as well of the principals of the selected schools.

The Disruptive Behavior Disorders (DBD) rating scale [31,32] was standardized for the populations of the Limpopo province of South Africa in an earlier study [14] and used as the screening instrument. Participation was voluntary. Informed consent was obtained from the child's parents or guardians. Both teachers and parents were given the rating scale to complete. Only the teacher's ratings were used for the screening, since the return of the parent's rating scale was below 50%, probably because many children either did not live with their parents or the parents were illiterate. Teacher ratings are usually regarded as an accurate measure of assessment [6]. The return of the teacher's rating scale was however close to 100%. The children meeting the criteria for inclusion into the groups with ADHD symptoms (~7%) were selected for further testing. They were matched for gender, age, and ethnic group with children who did not meet the inclusion criteria, obtained from the screening process.

Children were divided into a group with symptoms of ADHD and a comparison group without ADHD symptoms (Table 1), based on teacher ratings on the DBD rating scale [31,32]. Cut off points for the group with *ADHD *symptoms (95^th ^percentile or above) and *comparison group *(85^th ^percentile or below) were based on the results from the prevalence study [14] in which more than 6000 children in the Limpopo Province were rated on the DBD. According to the norms, scores on hyperactive/impulsive related items less than 15 *and *inattentive items less than 17 were regarded as *comparisons*. Scores higher than 18 on the Hyperactive/Impulsive items were classified as having symptoms of *ADHD-HI *and higher than 21 on the Inattention items were classified as having *ADHD-PI *symptoms. If the criteria were met on both types of items they were classified as having symptoms of *ADHD-C*.

**Table 1:** Sample characteristics

**Subtype**	**Afrikaans**	**Northern Sotho**	**Tsonga**	**Venda**	**North Ndebele**	**Tswana**	**Bolobedu**	**Total**
**ADHD-HI**								
*Boys*								
6–9 yr	6	5	1	2	1	3	5	23
10–13 yr	3	2	0	4	4	9	6	30
*Girls*								
6–9 yr	1	2	3	4	1	1	1	13
10–13 yr	2	1	2	0	1	0	2	8

**ADHD-PI**								
*Boys*								
6–9 yr	4	1	5	10	6	3	4	33
10–13 yr	4	3	3	5	3	2	8	28
*Girls*								
6–9 yr	1	4	3	1	1	1	2	13
10–13 yr	1	2	5	3	2	4	3	20

**ADHD-C**								
*Boys*								
6–9 yr	3	7	8	5	4	4	6	37
10–13 yr	4	6	7	6	4	6	5	38
*Girls*								
6–9 yr	3	2	1	2	2	1	2	13
10–13 yr	0	4	1	0	0	2	1	8

**Non-ADHD**								
*Boys*								
6–9 yr	13	13	14	17	11	10	15	93
10–13 yr	13	11	10	15	11	17	19	96
*Girls*								
6–9 yr	5	8	7	7	4	3	4	38
10–13 yr	3	7	8	3	3	6	7	37

**Total**	68	78	78	84	58	72	90	528

The final sample consisted of children from seven ethnic groups inhabiting the Limpopo Province (Table 1). Children with an IQ lower than 80 and/or with a history of neurological problems (e.g. epilepsy, head injuries, cerebral palsy, or cerebral malaria) were excluded. None of the children was on psychostimulant medication at the time of testing.

### Instruments

Assessment of, and research, on ADHD in developing countries like South Africa could be improved with standardized tests reliably differentiating between children with and without ADHD symptoms. The tests selected in the present study measure various aspects of fine motor functions, mainly assess basic non-verbal skills. They are simple, inexpensive, easy to transport to and use in remote rural areas without the conveniences of Western settings.

The three tests which measure different aspects of fine manual motor skills were the Grooved Pegboard Test [33] (distal, complex fine motor coordination and psychomotor speed), the Maze Coordination Task [33] (tactual motor coordination skills) and the Finger Tapping Test [34] (pure motor speed).

The *Grooved Pegboard *apparatus (Lafayette Instrument Company, #4202) consists of a metal board (10 × 10 cm) that contains a 5 × 5 set of holes each with a groove oriented randomly in different directions. Twenty-five round metal pegs with a ridge running lengthwise have to be rotated into the correct position for insertion into the holes. The child is instructed to insert the pegs as fast as possible, completing one row before starting on the next. The test is performed once with each hand, always starting with the dominant hand. Time to completion (in s) is the final score for each hand.

The following instruction is given in the child's own language:

"You are now going to put each of these pegs into the holes of this board (show). You can only use one hand. Pick up one peg at a time. Notice that the pegs are not round, neither are the holes in the board. In order to insert it you will have to rotate the peg so that it fits exactly (show two pegs, let the child try the three next, then remove all five pegs from the holes). When I tell you to start, you shall start over here (point to the upper left hole if the child is using its right hand and to the upper right hole if the child is using its left hand), fill this upper row, continue on the next, and so on until all the pegs are inserted. Try to be quick. Use only your (dominant/non-dominant) hand."

The *Maze Coordination Task *(Lafayette Instrument Company, #2706A) is a simple maze without blind alleys. The maze is placed at ~60 degree angle with the table. The child is required to go through the maze with an electric stylus, trying not to touch the sides. The stylus is connected to an electronic clock and a counter, which record the number of contacts the stylus is making with the sides (counter) and the cumulative time these contacts last (timer). The aim is to move the stylus through the maze, without touching the sides. There is no speed requirement. The test is performed twice with each hand. The total sum of touches and cumulative time of contact of two trials with the same-side hand are the final scores.

The following instruction is given in the child's own language:

*"In this test, take this stylus and move it through the maze all the way to here (point). Try to avoid touching the sides (show). Do this with about this speed. (Show by moving stylus through about 1/4 of the maze). You do not have to rush, if you move too quickly you will make more errors. Try to be accurate. Start with your (dominant) hand. Do not rest your hand or arm against anything"*.

The *Finger Tapping Test *apparatus (Marquardt, type 0925.0201) is a micro-switch operated by a key consisting of a metal arm and a round disk (20 mm in diameter). The key is placed at the short end of a 223 mm × 151 mm × 38 mm (h) plastic box where the operating hand is to be rested. The length of the metal arm from the micro-switch to the centre of the disc is 60 mm. The switch needs ~65 g dead weight to close. An electronic counter records the number of micro-switch closings (taps). The child has to press the switch ~15 mm to activate the counter. It is important that the hand is rested in a constant position in contact with the surface of the plastic box to ensure that only the index finger is moving. A stopwatch is used to time each 10-s trial. The child may rest at any time between trials, but is told to take a break at least after every third trial. For each hand, the test is terminated after ten trials, or when five consecutive trials do not vary by more than five taps. The means of the five trials with the highest number of taps are computed for each hand and used as the final scores.

The following instruction is given in the child's own language:

"Can you, please, show me how fast you can press this button with your (dominant) index finger? (If the child is small, touch the index finger. Demonstrate what the child has to do). Rest your arm in a comfortable position and try for yourself. You have to press the button all the way down and release it, or the counter will not work properly. Keep your wrist and arm still and remember to press as fast as you can. I will tell you when to start and when to stop."

### Procedure

The children were always tested by a tester fluent in the child's own language. Most assessments of motor functions of the South African children were done at their schools during school hours. The exceptions were the children whose school was within a radius of 2 km from the University and the children referred for assessment, which were tested at the University Clinic.

To assess hand dominance, the children were asked to hit a nail with a small hammer, throw a ball, and to write their name. Experimental tests were presented in the following order: Grooved Pegboard, Maze Coordination Task, and Finger Tapping Test. For the Afrikaans group, the IQ was established with the Senior South African Individual Scale (SSAIS) [35]. As there are no standardized IQ tests for the indigenous African populations, Raven's progressive matrices was used to estimate IQ [36,37]. This test is considered to be culture-fair [38]. The actual testing procedure for each child lasted about 45 min and was conducted by intern clinical psychologists.

### Statistical analysis

Raw scores were converted to standard scores (z-scores) for each ethnic group, to eliminate the effects of possible differences between testers and the translation of instructions. Group differences on demographic variables were analysed using analysis of variance (ANOVA) using the Statistica 6.1 programme [39]. The results were analysed twice with 4 × 2 × 2 (subtype × gender × hand dominance and subtype × age group × hand dominance) ANOVA's for independent samples, with dominant vs. non-dominant hand as within-child repeated measure. Post-hoc tests consisted of multiple comparisons using the Bonferroni correction.

## Results

### Analysis 1: gender effects

In general, children with high scores on the Disruptive Behavior Disorders (DBD) rating scales, who were classified as having ADHD symptoms, performed more poorly on the Grooved Pegboard and Motor Coordination Task than the comparison group without ADHD symptoms. Further, girls performed worse than boys. There were no differences in performance on the Finger Tapping Test.

**Figure 1:**
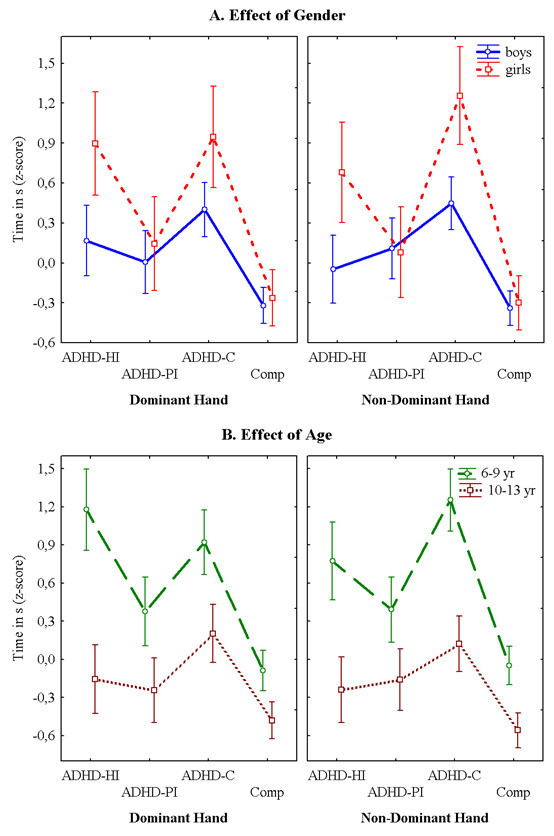
**Grooved Pegboard**. **A. **The graphs represents the means ± SEM of the time used by the groups with symptoms of the three ADHD subtypes and the non-ADHD comparison group to complete the grooved Pegboard, as a function of gender. The left side graph illustrates the time used with the dominant hand, while the one on the right side represents the time to complete the task with the non-dominant hand. **B. **Illustration of the means ± SEM of the time used by the groups with symptoms of the three ADHD subtypes and the non-ADHD comparison group to complete the Grooved Pegboard task as a function of age. The left side graph shows the performance with the dominant hand, while the right side graph shows the time taken with the non-dominant hand.

**Figure 2:**
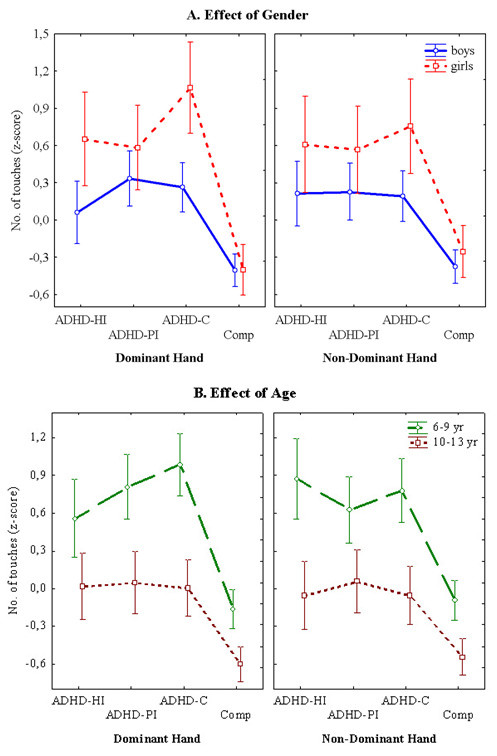
**Maze Coordination Task**. **A. **The graph shows the means ± SEM of number of touches against the sides of the maze made by the groups with symptoms of the three ADHD subtypes and the non-ADHD comparison group as a function of gender. The left side graph shows the performance with the dominant hand, while the right side graph shows the number of touches with the non-dominant hand. **B. **The graph shows the means ± SEM of the number of touches against the sides of the maze made by the groups with symptoms of the three ADHD subtypes and the non-ADHD comparison group as a function of age. The left side graph shows the performance with the dominant hand, while the right side graph shows the results with the non-dominant hand.

**Figure 3:**
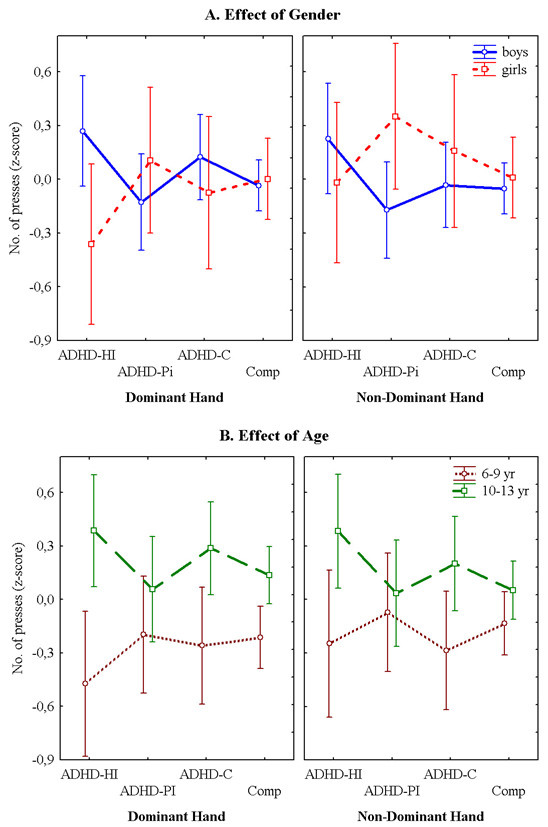
**Finger Tapping Test**. **A. **The means ± SEM of the number of presses are illustrated for the groups with symptoms of the three ADHD subtypes and the non-ADHD comparison group. The left hand graph shows the number of presses with the dominant hand while the right hand graph shows the performance with the non-dominant hand as a function of gender. **B. **The means ± SEM of the number of presses are depicted for the groups with symptoms of the three ADHD subtypes and the non-ADHD comparison group as a function of age. The left side graph shows the performance with the dominant hand and the right side graph with the non-dominant hand.

Figures 1A, 2A and 3A present the performances for each of the three tasks for both hands for the subtypes with ADHD symptoms and the non-ADHD comparison group for both genders. Figure 1A shows that the children with ADHD symptoms took more time than the comparisons to finish the task. This was the case for both the dominant and the non-dominant hand. Generally, the girls took longer to complete the task than the boys. This was however not the case for the group with Predominantly Inattentive symptoms (ADHD-PI). Figure 2A illustrates that the children with ADHD symptoms touched the side of the maze more often than the comparison group. The girls had overall more touches than the boys. This was the case with both hands. Figure 3A shows that there were no obvious differences in performance between the children with symptoms of the ADHD subtypes and the non-ADHD comparisons. There is also no obvious difference in performance between the genders.

The statistical results are shown in Table 2. For the Grooved Pegboard and the Maze Coordination Task there were statistically significant main effects of ADHD subtype and gender (ps < 0.001). On all three tests there was a statistically significant main effect of dominance (ps < 0.001).

**Table 2:** Results from repeated measures ANOVA

**Measure**	**Variable**	**ANOVA**		
			**Gender**	**Age**

**Grooved Pegboard**		**Df**	**F**	**F**
	ADHD Subtype	3, 512	14. 270***	40.936***
	Gender/Age	1, 512	2895.916***	105.042***
	Gender/Age × Subtype	3, 512	30.504**	5.216**
	Dominance	1, 512	47. 812***	0.308
	Dominance × Gender/Age	1, 512	0.328	0.206
	Dom × Subtype	3, 512	4. 790**	3.290*
	Dom × Gender/Age × Subtype	3, 512	2.285	3.373*

**Maze Coordination**				
	ADHD Subtype	3, 516	26.006***	37.571***
	Gender/Age	1, 516	13.694***	75.464***
	Gender/Age × Subtype	3, 516	1.672	2.244
	Dominance	1, 516	198.165***	0.050
	Dominance × Gender/Age	1, 516	0.045	0.055
	Dom × Subtype	3, 516	1.134	3.122*
	Dom × Gender/Age × Subtype	3, 516	0.414	2.729*

**Finger Tapping**				
	ADHD Subtype	3, 475	0.437	0.069
	Gender/Age	1, 475	2.584	19.961***
	Gender/Age × Subtype	3, 475	3.088*	1.596
	Dominance	1, 475	61.979***	0.304
	Dominance × Gender/Age	1, 475	8.290**	2.581
	Dom × Subtype	3, 475	0.589	0.493
	Dom × Gender/Age × Subtype	3, 475	1.572	0.120

*p < 0.05

** p < 0.01

*** p < 0.001

Post-hoc testing (with Bonferroni correction) of the Grooved Pegboard and the Maze Coordination Task showed that in both tests, the children with symptoms of the ADHD-C subtype had the poorest performance. The differences, when compared with the non-ADHD group, were statistically significant for both genders. Both girls and boys with symptoms of the ADHD-PI subtype performed significantly poorer than the comparison group only in the Maze Coordination Task with the dominant hand. This was the case for both genders. When girls with ADHD-HI symptoms used their dominant hand, they performed significantly worse than the boys on the Grooved Pegboard as well as the Maze Coordination Task.

### Analysis 2: age effects

Figures 1B, 2B and 3B present the performances for all three tasks for both hands for the children with symptoms of the ADHD subtypes and the non-ADHD comparison group for the age groups 6 – 9 yr and 10 – 13 yr. As could be expected, the younger group's performance was consistently poorer than the older group on all three tasks. Figure 1B shows that it was especially the children with symptoms of ADHD-C and ADHD-HI that encountered the most difficulties on the Grooved Pegboard. This was the case for both age groups for both hands. Figure 2B shows that on the Maze Coordination Task the children with symptoms of the ADHD subtypes' performance were poorer than that of the non-ADHD comparison group. This was true for both the dominant- and the non-dominant hand. The results were more pronounced in the younger group. Figure 3B illustrates that in both age groups there was little difference in finger tapping performance between the group with symptoms of the ADHD subtypes and the non-ADHD comparisons.

The effects of ADHD subtype symptoms, gender and age for all three tests are shown in Table 2. There were statistically significant main effects of symptoms of ADHD subtype (ps < 0.001) as well as a significant 3-way interaction effect between hand dominance × age × ADHD subtype symptoms (p = 0.018 and p = 0.043, respectively). On all three tests, there was also a statistically significant effect of age (p < 0.001).

Bonferroni corrected *post-hoc *tests on the Grooved Pegboard and the Maze Coordination Task showed that again, that the groups with symptoms of the ADHD-C subtype had the poorest performance. This was however, only in the case in the younger group. No significant effect was found in the difference between the older children with symptoms of ADHD-C and their non-ADHD comparisons. Independent of hand used, the younger children with symptoms of ADHD-HI differed significantly from the comparisons in the Grooved Pegboard, but not in the Maze Coordination Task. Similarly, independent of hand used, the younger children with ADHD-PI symptoms differed significantly from the comparisons on the Maze Coordination Task, but only with the non-dominant hand on the Grooved Pegboard. The older group (10 – 13) did not differ significantly, from the non-ADHD comparisons in any of the tasks.

## Discussion

The primary aim of the present study was to investigate if there is an association between symptoms of ADHD and motor skills, in African cultures also, as has already been implicated in Western studies [8,17,24,40,41] and whether these motor problems will differ as a function of ADHD subtype, gender, age, and hand dominance. The study compared South African children from seven different ethnic groups with symptoms of the three ADHD subtypes with a Non-ADHD comparison group on three measures of motor functioning: the Grooved Pegboard, which measures manual dexterity, complex coordination and movement speed; the Maze Coordination Task, which measures complex coordination, goal-directed fine movements, accuracy and stability of movement; and the Finger Tapping Test, which is a simple measure of finger movement and speed [42]. The tasks were performed with both the dominant and the non-dominant hands because when a task is performed with the non-dominant hand, it becomes more complex, probably because it requires continuous attention and more control [43,44].

### Association of motor problems with ADHD

The performance of all groups with symptoms of the three ADHD subtypes was significantly poorer on the Grooved Pegboard and the Maze Coordination Task than that of the non-ADHD comparisons. Maybe because the Finger Tapping Test is a simple measure of motor speed and does not involve complex coordination and goal-directed movements, there was no significant difference in performance between the children with ADHD symptomatology and the non-ADHD comparison group on this test. This result replicates that of a European study [45]. Deficits in motor control in ADHD have been reported previously especially when more complex motor sequences have to be performed [43]. Barkley [2] and Leung and Connolly [46] ascribe this to dysfunctional higher-order cognitive processes such as planning and behavioural organising, involved in the more complex motor tasks. However, not all researchers share this opinion. According to Sagvolden and co-workers [15,16] the neurobiological basis is predicted to be a hypofunctioning nigro-striatal dopaminergic system. Neuropsychological studies indicate that the areas involved in ADHD includes the basal ganglia, as well as the cerebellum and the prefrontal cortex [47].

The results of the present study show that the groups with ADHD symptoms were less impaired on the speeded task (Grooved Pegboard) than on the more complex Maze Coordination Task which requires more control, stability, and motor planning. The poor performance on the Maze Coordination Task indicated that children with ADHD symptoms appear to have problems with eye-hand coordination, and control of the task by means of prestructured motor plans [18] as this tasks requires planning ahead. The poorer performance on the Grooved Pegboard especially of the children with symptoms of ADHD-C, suggested that their eye-hand coordination is impaired when motor speed is required [48,49].

### Subtypes

All three subgroups showing symptoms of the ADHD had motor performance problems when compared to children without ADHD symptoms. The group with ADHD-C symptoms performed significantly poorer on both the Grooved Pegboard and the Maze Coordination Task, while there were no significant differences for the Finger Tapping Test. It is interesting to notice that the group with ADHD-PI symptoms only differed significantly from the comparison group on the Maze Coordination Task, but not on the Grooved Pegboard. An explanation may be that the Maze Coordination Task is slightly more complex than the Grooved Pegboard, which measures accuracy, and motor speed, but not the same degree of complex eye-hand coordination and motor planning as is required by the Maze Coordination Task.

Only the girls with ADHD-HI symptoms differed significantly from the comparison group in both the Grooved Pegboard and Maze Coordination Task. This was however only the case with the dominant hand. In general, the findings were in line with other studies which found the most pronounced impairment in the children with symptoms of ADHD-C and ADHD-PI subtypes [23,24,41,50-52]. An association between symptoms of inattention and poor motor skills is well-documented [17,23,41]. The study by Pitcher, Piek and Hay [41] found that 58% of children with ADHD-PI, 49% of ADHD-C, and 47% of ADHD-HI were having motor problems. The present study also supports the findings of Hinshaw and co-workers, using the Grooved Pegboard in girls with ADHD, that most impairments are found in the ADHD-C subtype with the ADHD-PI group impaired to a lesser degree [53]. This finding is remarkable as the scores on the Inattention scale of the DBD rating scale did not differ between the two groups (22.86 ± 2.65 vs. 22.84 ± 3.07) and a strong link between inattentiveness and motor dyscontrol has been reported in most studies [22,23,41]. A possible explanation may be that the additional hyperactivity/impulsiveness symptoms add to the impairment of children with symptoms of ADHD-C. The reason may be that poor fine motor skills make greater demands on sustained attention; therefore fine motor movements will be more affected in children with attention deficits than comparison children performing fine motor skills smoothly [41]. There is a strong association between inattention and movement difficulties, as a more pronounced inattention predicts more difficulties in motor coordination [23,41]. The lesser association of ADHD-HI symptoms with motor problems is also confirmed by most studies [41]. Impulsiveness has been associated with motor problems by Tseng and co-workers [54], their explanation was that impulsive children are more inaccurate and do not learn from their mistakes.

### Gender differences

Sex differences have only been infrequently assessed in the literature. Gaub and Carlson in their meta-analysis [55] found no difference in motor skills between the genders. In the present study, girls performed both the Grooved Pegboard and the Maze Coordination Task significantly poorer than the boys. This was however only the case with the dominant hand. This finding may support the statement by Biederman and co-workers [56] that, although ADHD is less frequent in girls, the symptoms are more severe than in boys.

### Age effect

Age was the most pronounced of the statistical effects. For all measures, except one, significant differences were only found in the younger group with symptoms of ADHD, when compared with the non-ADHD comparison group. The exceptions were the children with ADHD-C symptoms on the Grooved Pegboard. Independent of hand used, their performance was significantly poorer than that of the comparison group without ADHD symptoms. Some studies show that, although some children seem to outgrow their motor problems, they often persist into adulthood [57]. The results could be attributed to the effect of maturation on neuropsychological performance [21] and therefore the tasks could have been insensitive to differences in motor functioning between older children with and without symptoms of ADHD in the present study.

### Hand dominance

When the children with symptoms of ADHD showed significantly poorer performance with one hand only, it was the dominant hand. This was the case for both the boys and girls with ADHD-PI symptoms in the Maze Coordination Task and for only the girls with ADHD-HI symptoms on the Grooved Pegboard. This supports the findings of Kalff and co-workers [43] that children at risk for ADHD were disproportionately more inaccurate and had more unstable performance with their preferred hand than other children. The exception was the result of the younger boys with symptoms of ADHD-HI on the Maze Coordination Task where there was a significant poorer performance with the non-dominant hand when compared with their non-ADHD comparisons.

## Conclusion

This study shows that African children from different ethic groups with ADHD symptoms have poorer motor control, accuracy and speed when the tasks are fairly complex like in the Grooved Pegboard and Maze coordination Task. There was no observable difference when the task consists of a simple motor movement, like in the Finger Tapping Test. This deficiency in motor functioning is found in all ADHD subtypes, with the group with symptoms of ADHD-C most severely affected.

The study did generally not reveal striking gender differences; the exception was the significantly poorer performance of the ADHD-HI girls with the dominant hand on both the Grooved Pegboard and the Maze Coordination Task. The boys did not differ significantly from their non-ADHD comparisons when using the dominant hand. This result is difficult to explain, as it was only found in one ADHD subtype, but it cannot be attributed to chance, as it was observed in both the Grooved Pegboard and the Maze Coordination Task.

The results also showed that problems with motor control were less noticeable in the older groups, probably due to the effect of maturation, which made the tasks too easy for this particular age group.

Usually there were no differences between performances with the dominant and non-dominant hand when the children with ADHD symptoms were compared with the non-ADHD comparison group, the exception being the girls with ADHD-HI symptoms who performed significantly poorer with their dominant hand on two of the tasks than the non-ADHD girls. The younger boys on the other hand did have problems only on the Maze Coordination Task with their non-dominant hand.

The present study shows that problems with motor control in children with ADHD symptoms are not associated with culture or ethnicity, were present in all three ADHD subtypes, that both genders are affected, are less pronounced in older children, and that the problems exist in both hands. These are significant findings as children with motor problems are at risk for learning problems and poorer psychological adjustment [50,58]. Because motor clumsiness is not a diagnostic criteria in DSM-IV [1], it is often not assessed when ADHD is diagnosed and the child may go without intervention.

## Abbreviations

ADHD – Attention-Deficit/hyperactivity Disorder

ADHD-C – ADHD (Combined subtype)

ADHD-HI – ADHD (Hyperactive/impulsive subtype)

ADHD-PI – ADHD (Predominantly Inattentive subtype)

DBD – Disruptive Behavior Disorders rating scale

## Competing interests

The author(s) declare that they have no competing interests.

## Authors' contributions

AM participated in the development of the study design, supervised the data collection, prepared the data, performed the statistical analysis, and wrote the manuscript.

TS participated in the development of the study design, worked on the manuscript and approved the final draft.

**Table 3:** Comparing ADHD subtypes with non-ADHD groups

	**ADHD-HI**		**ADHD-PI**		**ADHD-C**	
***Gender groups***						

**Measure**	**Boys**	**Girls**	**Boys**	**Girls**	**Boys**	**Girls**

*Pegboard*						
Dom. hand	n/s	0.006**	n/s	n/s	0.001**	0.002**
Non-dom.	n/s	n/s	n/s	n/s	0.000***	0.000***
*Maze*						
Dom. hand	n/s	0.037*	0.003**	0.032*	0.004**	0.000***
Non-dom.	n/s	n/s	n/s	n/s	0.04*	0.04*

***Age groups***						

**Measure**	**6 – 9**	**10 – 13**	**6 – 9**	**10 – 13**	**6 – 9**	**10 – 13**

*Pegboard*						
Dom. hand	0.000***	n/s	n/s	n/s	0.000***	0.008**
Non-dom.	0.032*	n/s	n/s	n/s	0.000***	0.008**
*Maze*						
Dom. hand	n/s	n/s	0.000***	n/s	0.000***	n/s
Non-dom.	0.000***	n/s	0.04*	n/s	0.001**	n/s

*p < 0.05

** p < 0.01

*** p < 0.001

With Bonferroni corrections.

There were no significant differences when the ADHD subtypes were compared with each other, with one exception: The performance with the non-dominant hand on the Grooved Pegboard of the younger ADHD-PI subtype differed significantly from the ADHD-C subtype (p = 0.03)
